# Beyond Access: Behavior Change and Gendered Realities in the Adoption of Digital Health Tools for Maternal, Newborn, and Child Health in Nigeria

**DOI:** 10.21106/IJMA_45_2025

**Published:** 2026-01-29

**Authors:** Charles Chibuisi Ehiemere, Ishaku Adamu Akyala, Bisayo Adetuyi Ogundipe, Chukwuebuka Clement Nri-Ezedi, Peter Joseph Anzaku, Waryit Esther Yonah, Efe Mary Imokhai, Isaac Onyebuchi Okohu, Idowu Olapemi Makinde, Ngozi Miriam Chimzobam-Nnalue

**Affiliations:** 1 Department of Public Health, Global Health and Infectious Diseases Control Institute, Nasarawa State University, Keffi, Nigeria.; 2 Global Health and Infectious Diseases Control Institute, Nasarawa State University, Keffi, Nigeria.; 3 Department of Health Economics, Management and Policy, Global Health and Infectious Diseases Control Institute, Nasarawa State University, Keffi, Nigeria.

**Keywords:** Behavior Change, Digital Health, Gender, Health-Seeking Behavior, Maternal and Child Health, Nigeria, Technology adoption

## Abstract

**Background and Objective:**

Digital health innovations are increasingly being deployed to improve maternal, newborn, and child health (MNCH) outcomes in Nigeria. However, their adoption and effectiveness are influenced not only by technological access and infrastructure but also by behavioral factors, gender norms, and cultural contexts that shape user engagement and health-seeking behaviors.

**Methods:**

A narrative review was conducted using PubMed, Web of Science, Scopus, and African Journals Online, covering studies published between 2015 and 2025. From an initial pool of records, 46 studies met the inclusion criteria following screening. This review synthesized empirical studies, program evaluations, and behavioral frameworks, such as the Fogg Behavior Model and Health Belief Model, to examine how gender dynamics, social hierarchies, and behavioral readiness shape digital MNCH adoption.

**Results:**

The findings reveal that digital literacy gaps, gendered power relations, sociocultural beliefs, and intra-household decision-making processes significantly affect technology uptake. Interventions that incorporated voice-based content, local languages, and male-inclusive engagement strategies demonstrated higher levels of acceptance and retention in the target population. Behaviorally informed designs, culturally tailored messaging, and the involvement of trusted intermediaries, such as community health workers, were critical success factors.

**Conclusion and Global Health Implications:**

Effective digital health interventions must move beyond mere technical provision to integrate behavioral science and gender responsiveness into their design and delivery. Approaches that prioritize the lived realities of users, particularly women’s agency within household decision-making, are more likely to achieve equitable and sustainable MNCH outcomes. Policy and program developers should prioritize human-centered, behaviorally intelligent, and gender-transformative digital health models, while future interventions must integrate cultural adaptability, motivational triggers, and inclusive engagement strategies to enhance digital MNCH impact

## INTRODUCTION

Digital health technologies, such as short message service (SMS) reminders, mobile applications, teleconsultations, and interactive voice response (IVR) systems, are increasingly deployed to address maternal, newborn, and child health (MNCH) challenges in Nigeria.^[1–4]^ These tools offer great promise for improving access, coverage, and health education, particularly in underserved communities. However, the availability of technology does not guarantee its adoption. Utilization is shaped by behavioral triggers, individual motivation, and social and gender dynamics.^[5,6]^

Rural infrastructure limitations, including weak mobile networks and unreliable electricity, further hinder digital health deployment.^[7,8]^ However, beyond infrastructure, gender remains a critical and often overlooked factor. Women face barriers such as limited literacy, mobility constraints, low phone ownership, and decision-making dynamics that are dominated by men.^[9–13]^ In many Nigerian households, men’s control over resources, including phones and health decisions, undermines women’s ability to access or act on health messages.^[14,15]^ Even the design of digital interventions can reinforce exclusion if they fail to account for these realities.^[12,16]^

Economic and educational disadvantages further exacerbate this problem. Uneducated, unemployed women, or those living in rural areas, face greater difficulty accessing digital health services, reflecting a wider trend across Sub-Saharan Africa.^[17–19]^ Societal norms that prioritize male ownership and gatekeeping often extend into digital domains, restricting women’s participation.^[20]^ Closing this gap requires more than technology; it demands behaviorally intelligent and gender-responsive design. When integrated effectively, digital health tools can reduce disparities and enhance access to quality care for patients. Achieving this depends on culturally adapted design, inclusive messaging, and ethical implementation strategies that prioritize users’ lived realities.^[21,22]^

This study explores how gender dynamics and behavior change intersect in digital health adoption for MNCH in Nigeria. Through a narrative synthesis of evidence, it offers actionable insights into policy, practice, and design.

## LITERATURE REVIEW

In Nigeria, deeply rooted gender norms continue to shape MNCH outcomes. Patriarchal structures limit women’s autonomy in health-related decisions, particularly in rural and northern regions, where male partners or senior family members often control decisions on antenatal care, contraception, and child immunization.^[23,24]^ This power asymmetry extends to the adoption of digital health tools, constraining women’s ability to engage with platforms designed to support their health care. Similar dynamics have been observed in other Sub-Saharan contexts. In Zambia, for example, men often control maternal health decisions despite a limited understanding of health needs.^[25]^ Gendered cultural practices, such as naming conventions, reinforce structural inequalities.^[26]^ In Kogi State, Nigeria, certain norms impose indirect costs or social pressure, deterring women from seeking skilled maternal care.^[27]^

Lack of women’s autonomy is linked to poor MNCH outcomes, with evidence from Tanzania showing that greater decision-making power improves access to contraception and health services.^[28]^ In Nigeria, early marriage, low education, and entrenched cultural norms, especially in rural areas, reduce antenatal care utilization.^[29]^ Socioeconomic, religious, and regional factors, along with conflict, further limit immunization coverage.^[30]^ Addressing these barriers requires culturally sensitive strategies that expand education, health insurance, and economic empowerment and enhance autonomy and gender equity.^[27]^ Digital health mirrors these inequalities, as some women often require permission to use devices, face literacy gaps, and prefer trusted community-endorsed tools.^[12,31]^ Engaging men as allies in digital health can improve maternal outcomes, adherence, and uptake.^[32]^

### Behavioral Drivers of Digital Health Use

The adoption of digital MNCH tools in Nigeria depends as much on behavioral readiness as on availability, with key drivers including perceived vulnerability, self-efficacy, access, and supportive social norms. The health belief model (HBM) highlights that action is more likely when benefits outweigh barriers; however, many women face spousal disapproval and cultural taboos,^[33]^ while the Fogg behavior model (FBM) shows that even motivated users may not engage without adequate ability or prompts.^[34]^ Evidence from platforms such as Hello Mama and Mobile Midwife reveals that household power dynamics and fear of stigma hinder use,^[12]^ whereas male-inclusive, locally tailored, and audio-based interventions can significantly improve uptake, particularly among low-literacy users.

### Role of Community Health Workers (CHWs) and Social Networks

CHWs play an indispensable role in bridging the digital and behavioral divides. When digital platforms were coupled with interpersonal counseling, such as with the Safe Delivery App, outcomes improved in terms of knowledge retention and service uptake. CHWs enhanced trust, clarified digital content, and reinforced behavioral prompts, particularly in rural areas.^[34]^ Peer support networks have also proven to be effective. In areas lacking formal health communication infrastructure, social learning and peer-driven platforms serve as credible channels for reinforcing MNCH messages and encouraging digital adoption.^[35]^

### The FAMILIA Platform: A Behavioral and Gender-Responsive Model

FAMILIA is a digital health platform for MNCH in Nigeria that applies the capability, opportunity, motivation-behavior (COM-B), FBM, and HBM frameworks to enhance capability, opportunity, motivation, and readiness for technology adoption. It offers culturally relevant, stage-based content through multilingual IVR, unstructured supplementary service data (USSD)/SMS, and local-language storytelling, with features such as offiine access, cues to action, and male partner engagement to reach digitally marginalized users. Community influencer involvement further fosters motivation, household dialogue, and social reinforcement. This gender-responsive, context-adapted design moves beyond information delivery to embed sustainable behavior change mechanisms and address gender inequities. Table 1 outlines the behavior change models underpinning FAMILIA, their key constructs, and their application to digital MNCH adoption in Nigeria.

**Table 1:** Behavior change theories and application to digital MNCH.

Model	Key Constructs	Application to MNCH Digital Adoption
Health Belief Model (HBM)	Perceived severity, benefits, barriers, cues to action	Local-Language reminders on ANC visits, risk framing for missed care
Fogg Behavior Model (FBM)	Motivation, Ability, Prompt	IVR reduces literacy barriers; timed prompts via SMS/CHWs
COM-B	Capability, Opportunity, Motivation	Enables use via feature phones, emotional triggers around child milestones
Transtheoretical Model (TTM)	Stages of change	Content sequences for pregnancy to postpartum stages

## METHODS

This study adopts a narrative review methodology to evidence on behavior change and gendered adoption of digital MNCH tools in Nigeria. Unlike systematic or scoping reviews, narrative reviews provide flexibility in exploring diverse sources of peer-reviewed literature, grey documents, implementation reports, and theoretical publications.

We conducted a narrative review across PubMed, Web of Science, Scopus, and African Journals Online (AJOL) (2015– 2025), using key terms such as *“digital health”, “behavior change”, “maternal health”, “gender”, “Nigeria”*, and *“mobile health adoption.”* From 230 records, 46 met the criteria after double-review screening and snowballing to capture key theoretical and empirical studies. To comply with the IJMA’s 35-reference limit, we refined our selection to retain only the most relevant evidence. Data were thematically synthesized, and limitations, including narrative design and author involvement in the FAMILIA model, are acknowledged.

Snowballing techniques were employed to identify influential theoretical and empirical studies, especially those grounded in the HBM, FBM, diffusion of innovation, technology acceptance model, and COM-B frameworks.

### The Selection Criteria Included

- Relevance to digital health and MNCH outcomes
- Explicit reference to behavioral or gender factors
- Published from 2015 to 2025, with a focus on sub-Saharan Africa and low- and middle-income countries.

Data were analyzed thematically using an interpretive synthesis approach to identify converging patterns, key barriers, enabling features, and theoretical alignments.

The FAMILIA platform, developed during the author’s doctoral research, is a conceptual, co-designed tool applying behavioral and gender-responsive frameworks (COM-B, FBM, HBM) to digital MNCH design in Nigeria. Informally tested with stakeholders and end users, it awaits formal piloting. Here, it serves as an illustrative theory-to-design example, with conflict disclosed.

## DISCUSSION

Nigeria’s expanding digital health initiatives often underperform due to the “Retention-Readiness Gap”, where behavioral science is insufficiently integrated, leading to low retention and impact of the initiatives. Models such as the FBM, COM-B, and HBM show that access and prompts are ineffective without motivation, trust, and perceived benefits. Gender-related barriers, such as limited device ownership, male gatekeeping, and unequal decision-making, further restrict women’s participation. Male-inclusive interventions, such as FAMILIA’s targeted features, can address these challenges by fostering shared decision-making and supporting lasting behavior change.

**Figure 1:**
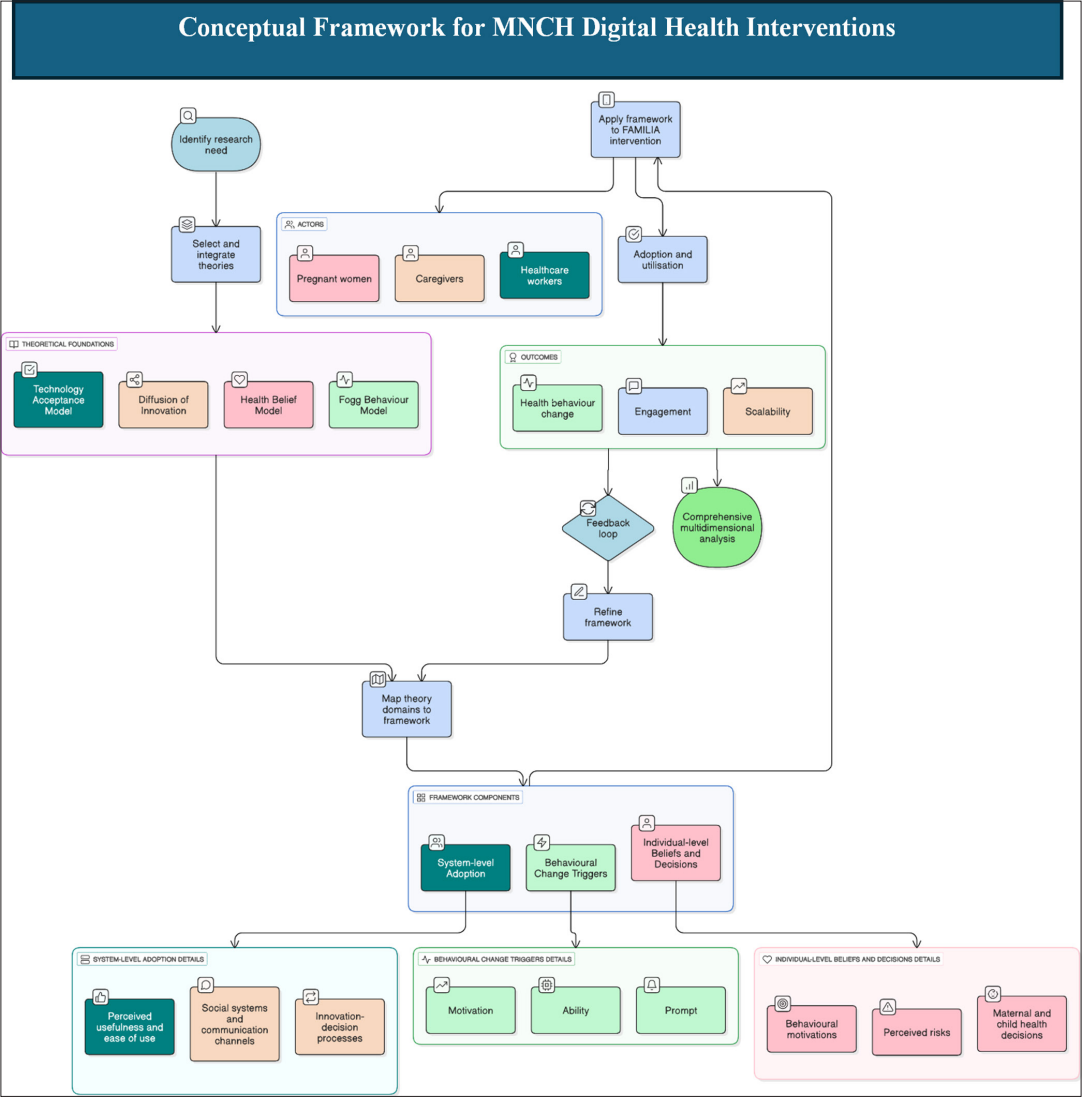
Conceptual framework for digital health integration in maternal, newborn, and child health services. The framework was developed by EHIEMERE Charles Chibuisi, drawing upon the technology acceptance model, digital object identifier, Health Belief Model, and Fogg Behavior Model, to guide the study.

Although not yet field-tested, the FAMILIA digital platform represents a best-practice model of theory-to-design translation. As illustrated in Figure 1, Tables 2 and 3, its features align directly with FBM principles.

**Table 2:** Illustrating targeted behavior change domains using the Fogg Behavior Model (FBM)-behavior occurs when motivation, ability, and prompt converge.

Target Group	Desired Behavior	Motivation (Why?)	Ability (Can?)	Prompt (Trigger)
Mothers/Caregivers	Attend ANC early & consistently	Health of baby & self	Transport, low cost	CHW visit, IVR/SMS reminders
Family decision-makers	Approve facility-based delivery	Safety & cost-benefit	Knowledge, transport access	Male-involvement sessions
Health workers	Follow neonatal care protocols	Saving lives, recognition	Equipment, skills training	Supervision, digital prompts
Community leaders	Promote immunisation campaigns	Protect children, trust	Mobilization support	Community dialogue/ Town hall

**Table 3:** Behavior change design features of familia solutions platform using the fogg behavior model, an Illustration of how familia applies motivation, ability, and prompts across MNCH user groups to support digital tool adoption.

Motivation	Ability	Prompt
Emphasize benefits to emotionally resonating messages	Accommodate Interactive Voice response (IVR) in local languages	Send reminders and appointment and health practices
Educate Healthcare workers provide rationale of digital health benefits	Prepare healthcare workers training and educational support	Engage Healthcare workers alert for client follow up
Encourage mate partners highlighting its positives in family well-being	Ease access for community leaders’ basic phones	Involve male partners and Community leaders via community meeting as advocates

Motivation: Emotional and cultural resonance through local-language audio storytelling and health benefit framing.

Ability: Reduced friction through IVR, USSD, and no-cost access through basic phones.

Prompt: Timed reminders, CHW nudges, and milestone-linked triggers. This approach embodies “behavioral engineering”, not simply delivering information but designing user pathways that frame action.

Digital MNCH interventions in Nigeria face both infrastructure gaps and weak behavioral integration, which limit their adoption. Undertrained health workers, low motivation, and poor user orientation compound these challenges. Embedding tools in primary care and leveraging CHWs with FBM-guided strategies can align motivation, ability, and prompts to drive lasting behavior changes [Tables 2 and 3].

CHWs, traditional birth attendants, and religious leaders are crucial for building trust and contextualizing digital health messages, serving as both prompts and reinforcements to drive behavioral change. However, many MNCH interventions in Nigeria remain donor-dependent, poorly integrated with national systems, and lack sustainability plans or feedback loops. Trust-building through respected local intermediaries enhances legitimacy and adoption, making it as critical as usability in the design. This review calls for behaviorally intelligent digital health policies developed from the outset of policy and funding, prioritizing gender responsiveness, digital literacy, motivational alignment, and culturally relevant triggers. Monitoring must track behavioral outcomes, such as antenatal care completion, male support, and immunization adherence, to ensure that interventions have a lasting impact.

While this review highlights the critical behavioral and gendered dimensions of digital MNCH adoption, it is not without limitations. As a narrative review, it may introduce selection bias and limit reproducibility compared to systematic reviews. The final inclusion of 35 studies reflected both relevance and journal requirements. In addition, the author’s involvement in the FAMILIA model may have introduced bias, although every effort was made to maintain objectivity.

## RECOMMENDATIONS

### Policy

The Federal Ministry of Health should develop national guidelines that explicitly incorporate behavioral science frameworks into the design, implementation, and evaluation of digital health interventions.

### Programmatic

1. Programs must ensure that content is available in local languages, delivered through low-literacy-friendly formats (e.g., IVR), and actively involve male household members.
2. CHWs should be trained in behavioral principles to reinforce digital messaging and support sustained user engagement.
3. Participatory co-creation with users across different stages of behavioral readiness is essential to guarantee relevance, usability, and long-term scalability of interventions.

### Research

Digital health interventions should integrate behavioral science frameworks (e.g., FBM, COM-B, HBM) from the outset to guide content development, delivery formats, and engagement strategies.

## CONCLUSION AND GLOBAL HEALTH IMPLICATIONS

Digital health holds significant promise for improving MNCH outcomes in Nigeria, but its impact is constrained without attention to behavioral and gendered adoption dynamics. Sociocultural norms, gender power imbalances, and motivational readiness critically shape the uptake of these services. Integrating behavioral science frameworks such as the FBM, COM-B, HBM, and the transtheoretical model offers pathways for designing persuasive, transformative tools. While innovations like FAMILIA remain untested in the field, they exemplify people-centered, gender-responsive, and behaviorally intelligent design. To unlock the full potential of digital health, Nigeria must institutionalize supportive policies and systems that empower individuals, particularly women, to act on health knowledge and sustain positive health behaviors.

### Key Messages

(1) Digital health tools are not inherently empowering; their adoption is significantly influenced by gender norms and socio-cultural structures. (2) Behavior change frameworks are critical for ensuring the sustained use of digital tools for maternal and child health. (3) Inclusive and gender-sensitive designs involving both men and women enhance digital health outcomes in low-resource settings.

## Acknowledgments

None.

## COMPLIANCE WITH ETHICAL STANDARDS

### Conflicts of Interest

The first author is the developer of the FAMILIA solutions digital health platform referenced in this article. This has been disclosed for transparency. While FAMILIA Solutions was co-designed with stakeholders and informally tested, formal piloting with ethical clearance is pending. The co-authors declare no conflicts of interest.

### Financial Disclosure

Nothing to declare.

### Ethics Approval

Not applicable.

### Declaration of Patient Consent

Patient’s consent is not required as there are no patients in this study.

### Use of Artificial Intelligence (AI)-Assisted Technology for Manuscript Preparation

The authors confirm that there was no use of artificial intelligence (AI)-assisted technology for assisting in the writing or editing of the manuscript and no images were manipulated using AI.

### Disclaimer

None.
